# Assessment of Knowledge, Attitude, and Practice Regarding Colorectal Carcinoma Screening Among Healthcare Personnel

**DOI:** 10.7759/cureus.64574

**Published:** 2024-07-15

**Authors:** Ahmad Khan, Hakim U Wazir, Muhammad Javed, Hamayoun Khan, Luqman Khan, Muhammad Aasim Khan, Arshad Khan

**Affiliations:** 1 College of Medicine, Gajju Khan Medical College, Swabi, PAK; 2 Gastroenterology, Gajju Khan Medical College, Swabi, PAK; 3 Gastroenterology and Hepatology, Gajju Khan Medical College, Swabi, PAK

**Keywords:** fecal occult blood test, healthcare personnel, colorectal carcinoma, practice, attitude, knowledge

## Abstract

Background: Colorectal carcinoma (CRC) is a rising issue worldwide, with high morbidity and mortality rates. It is the third most common cause of death globally. Early diagnosis can lead to prevention and treatment, making it crucial for healthcare professionals to have proper knowledge about CRC screening.

Aims and objectives: This study aimed to assess the level of awareness, identify knowledge gaps, and update the knowledge of healthcare workers.

Methods: This descriptive cross-sectional study was conducted from May to October 2023, in multiple tertiary care hospitals of Khyber Pakhtunkhwa, Pakistan. Responses were collected manually through a designed proforma of questionnaires.

Results: A total of 164 participants (137 male and 27 female) took part in our study. Among the participants, 92.1% (n = 151) were aware that colonoscopy is used for CRC screening. Other screening methods known to them included a fecal occult blood test (FOBT) (65.9%, n = 108), flexible sigmoidoscopy (48.2%, n = 79), stool DNA test (31.1%, n = 51), and virtual colonoscopy (34.1%, n = 56). Only 6.1% (n = 10) routinely recommended CRC screening for all patients, 22.6% (n = 37) recommended it occasionally, and 71.3% (n = 117) rarely or never recommended it. Regarding factors influencing the recommendation of CRC screening, 83.5% (n = 137) cited family history of CRC as the major factor, followed by patient age (68.3%, n = 112), availability of screening facilities (46.3%, n = 76), patient's overall health status (37.2%, n = 61), and patient's preference (20.7%, n = 34).

Conclusion: This study concluded that only a small proportion of healthcare personnel regularly recommend CRC screening. In addition, a small proportion are familiar with CRC screening guidelines, although most are well-informed about the various investigations used for screening.

## Introduction

Colorectal carcinoma (CRC) arises from uncontrolled and excessive cell growth within the large intestine, sometimes classified separately as rectal and colon cancer. It develops slowly, with a tumor doubling time of approximately 200 days, but can metastasize to adjacent and distant tissues. About 95% of CRCs originate from glandular cells in the large bowel wall and are called adenocarcinomas. The rectosigmoid region is the most common site, although other parts of the colon can also be involved. Colon cancer usually begins as benign polyps on the colonic mucosa, with about 5% potentially becoming malignant over time [1]. CRC is the third most commonly diagnosed cancer and the second leading cause of mortality worldwide. In 2020, 1.9 million new cases of colorectal cancer were diagnosed, and 0.9 million deaths occurred globally [2]. The highest prevalence rates were observed in New Zealand, Australia, and Europe (40.6 per 100,000 males), and the lowest in several Southern Asian and African regions (4.4 per 100,000 females). Mortality rates followed a similar pattern, with the highest in Eastern Europe (20.2 per 100,000 males) and the lowest in Southern Asia (2.5 per 100,000 females) [3]. By 2040, new global CRC cases are projected to rise to 3.2 million [2]. In Malaysia, CRC is the second most common cancer, accounting for 13.2% of all cancer cases, and the most common cancer among men, with an incidence rate of 16.3% [4]. In Pakistan, according to the collective registry report on December 2022, the Shaukat Khanum Memorial Cancer Hospital and Research Center reported CRC as the second most common cancer in Pakistan, among the top 10 malignancies diagnosed at the hospital during 28 years from December 1994 to December 2022 [5].

Multiple factors contribute to the development of CRC. Those with a family or genetic history of CRC are at a higher risk. Other risk factors include cigarette smoking, alcohol consumption, familial adenomatous polyposis (FAP), Lynch syndrome, inflammatory bowel disease (IBD), hereditary non-polyposis colorectal cancer, cholecystectomy, diabetes mellitus, and certain dietary patterns, such as low fiber intake, low consumption of vegetables and fruits, high intake of processed red meat, and constipation [1,6]. According to the American Cancer Society, men have about a 31% greater chance of developing CRC than women [7]. Colorectal cancer screening is recommended by various gastrointestinal (GI) societies for men and women at average risk, starting at age 50. Individuals at average risk have no family history of CRC, no inflammatory bowel disease, no inherited syndromes like Lynch syndrome, and no prior colon cancer diagnosis. Recommended tests include flexible sigmoidoscopy every five years (or every 10 years with fecal immunochemical test (FIT) or fecal occult blood test (FOBT)), colonoscopy every 10 years, double-contrast barium enema (DCBE) every five years, CT colonography, stool guaiac test (a type of FOBT) annually, stool DNA test as recommended by a doctor, and FIT annually. The US Preventive Services Task Force (USPSTF) suggests routine screening for individuals aged 45 to 75, without specifying a particular method. For adults aged 76 to 85, screening decisions should be personalized based on discussions with their physicians. The American Cancer Society also recommends starting routine screening at age 45 due to rising CRC incidence in younger people. Any abnormal findings from these tests should be followed by a colonoscopy [8].

Globally, CRC is a major public health problem, ranking as the third most common cancer and the second leading cause of cancer-related deaths. Early detection through screening can significantly reduce the death rate. Effective implementation of screening depends heavily on clinicians' knowledge and commitment to CRC screening recommendations. The purpose of this study was to survey the knowledge, attitudes, and screening practices of doctors working in tertiary care facilities in Khyber Pakhtunkhwa (KP), Pakistan.

## Materials and methods

This descriptive cross-sectional study was conducted in multiple tertiary care hospitals in KP, Pakistan, including Khyber Teaching Hospital Peshawar (KTH Peshawar), Hayatabad Medical Complex (HMC), Mardan Medical Complex (MMC), Bacha Khan Medical Complex Swabi (BKMC Swabi), and Saidu Group of Teaching Hospital (SGOH). The study population consisted of healthcare personnel, including house officers, medical officers, trainee medical officers, associate professors, assistant professors, and professors. A random sampling method was used, with a sample size calculated using the RaoSoft sample size calculator (RaoSoft Inc., Washington, USA). For a population of 2,116 and an anticipated frequency of 9% (according to a study conducted in Karachi, Pakistan), with a 95% confidence interval, the required sample size was at least 119 participants [9]. The study was conducted over six months, from May to October 2023. The study was approved by the Institutional Review Committee of Medical Teaching Institution, Gajju Khan Medical College/Bacha Khan Medical Complex, Swabi, Pakistan (reference no.: 2254/Ethical Board/GKMC dated June 15, 2023).

The study included healthcare personnel from the Medicine and Surgery departments, focusing on their perspectives and practices related to CRC screening. Personnel from other departments were excluded to maintain the study's focus on specialties directly involved in patient care and screening practices relevant to CRC. Data collection was carried out using a self-structured questionnaire composed of 16 questions (see Appendix), distributed both online and offline (predominantly in hard copy). The questionnaire was divided into the following sections: demographic data and place of practice, knowledge of CRC screening modalities, practice of CRC screening, and perceived barriers affecting decision-making regarding CRC screening. The questions were straightforward, concise, unambiguous, and presented in a logical order. Respondents filled out the data collection form directly. Participants provided consent before completing the questionnaire. Data were analyzed using IBM SPSS Statistics for Windows, version 26.0 (released 2019, IBM Corp., Armonk, NY). Descriptive analyses were conducted to summarize sample characteristics and assess CRC screening knowledge, attitudes, and practices among physicians. Sociodemographic variables were analyzed and presented as frequencies and percentages.

## Results

**Figure 1:**
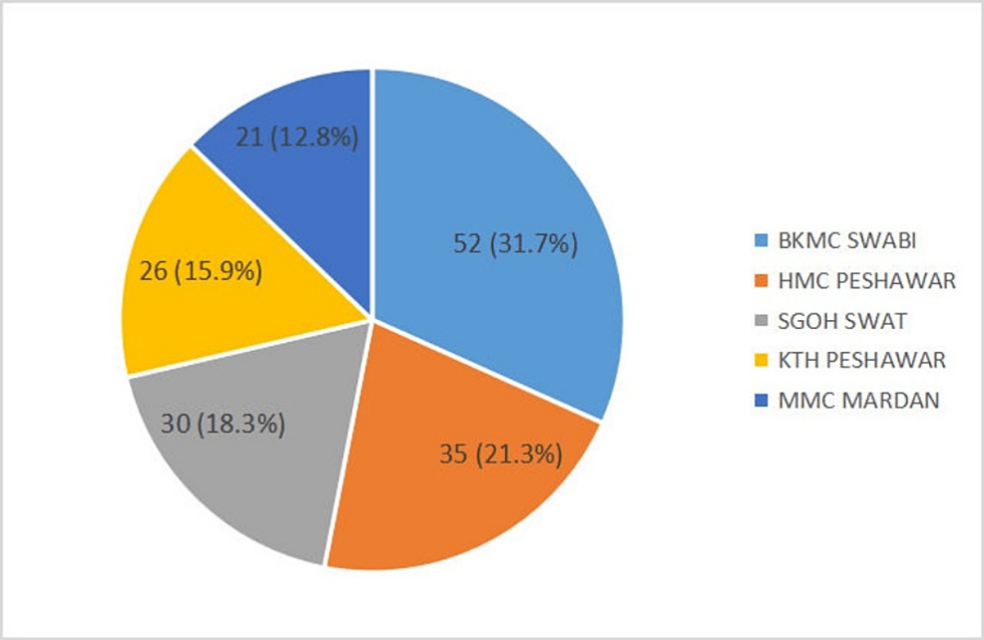
Distribution of participants by hospital Bacha Khan Medical Complex Swabi (BKMC Swabi), Hayatabad Medical Complex Peshawar (HMC Peshawar), Saidu Group of Teaching Hospital Swat (SGOH Swat), Khyber Teaching Hospital Peshawar (KTH Peshawar), Mardan Medical Complex Mardan (MMC Mardan)

A total of 164 participants were included in our study; 137 (83.5%) were males and 27 (16.5%) were females. The sample population was comprised of six professors, 16 associate professors, 22 assistant professors, 57 trainee medical officers (TMOs), 48 house officers (HOs), and 15 medical officers (MOs). The distribution of the participants by hospital was as follows: 52 (31.7%) from BKMC Swabi, 35 (21.3%) from HMC Peshawar, 26 (15.9%) from KTH Peshawar, 21 (12.8%) from MMC Mardan, and 30 (18.3%) from SGOH Swat (Figure 1).

**Figure 2:**
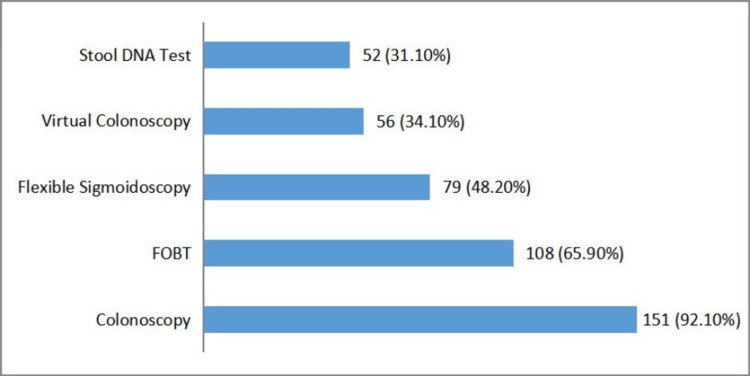
Frequency of screening methods for colorectal carcinoma FOBT: fecal occult blood test

About 151 (92.1%) were aware that colonoscopy is used for CRC screening. Awareness of other screening methods included FOBT with about 108 (65.9%), flexible sigmoidoscopy with about 79 (48.2%), stool DNA test with 51 (31.1%), and virtual colonoscopy with 56 (34.1%) (Figure 2).

**Figure 3:**
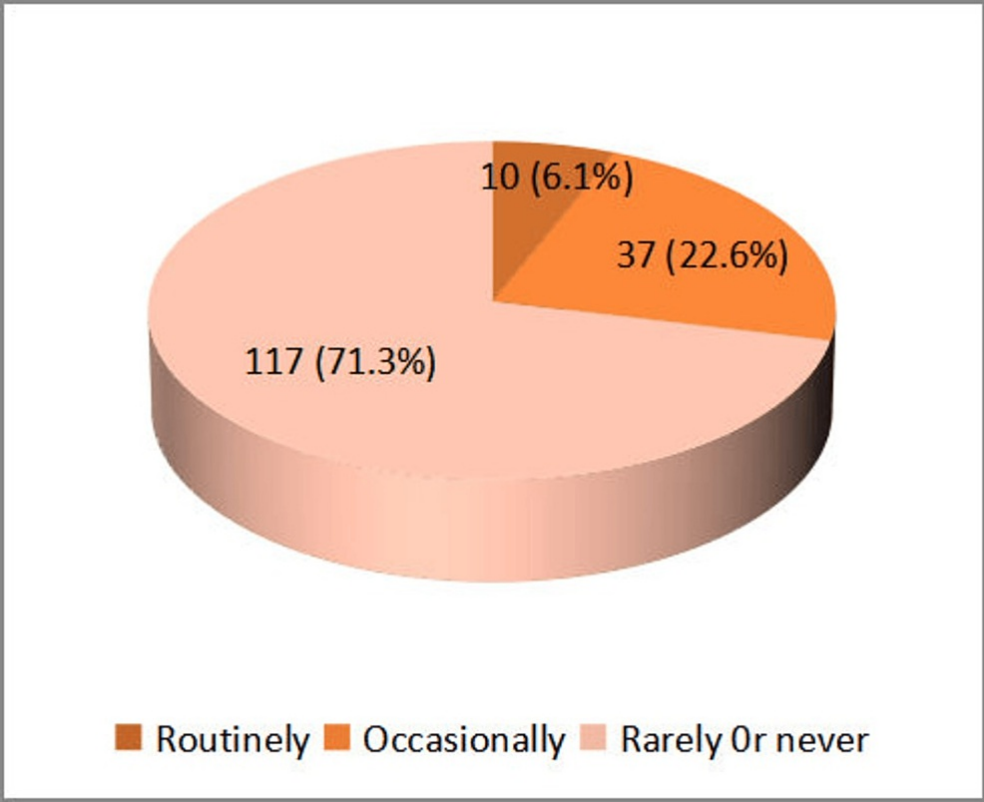
Frequency of recommendation for colorectal carcinoma (CRC) screening

Only 10 (6.1%) participants reported that they routinely recommend CRC screening for all patients, 37 (22.6%) recommend it occasionally, and 117 (71.3%) rarely or never recommend it (Figure 3).

**Figure 4:**
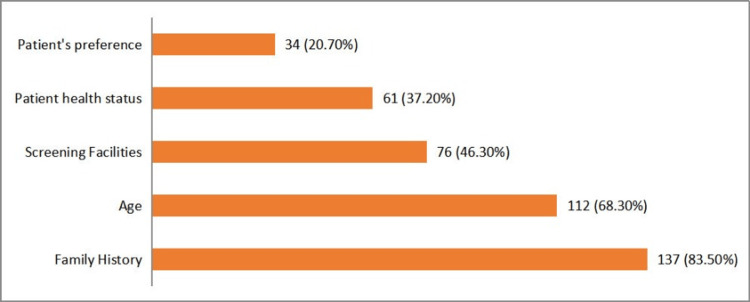
Factors influencing colorectal carcinoma (CRC) screening

The major factor influencing CRC screening recommendations was a family history of CRC, cited by 137 (83.5%) participants. Other influential factors included the patient's age (112, 68.3%), availability of screening facilities (76, 46.3%), the patient's overall health status (61, 37.2%), and the patient's preference (34, 20.7%) (Figure 4).

**Figure 5:**
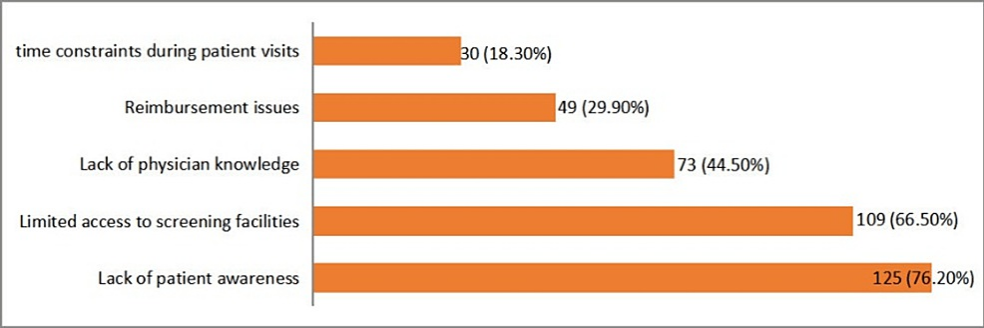
Major barriers in colorectal carcinoma (CRC) screening

Approximately 27 (16.5%) participants were familiar with CRC screening guidelines, 32 (19.5%) were somewhat familiar, and 105 (64%) were not familiar at all. Major barriers to CRC screening identified by the participants included lack of patient awareness (125, 76.2%), limited access to screening facilities (109, 66.5%), lack of physician awareness (73, 44.5%), reimbursement issues (49, 29.9%), and time constraints during patient visits (30, 18.3%) (Figure 5).

## Discussion

CRC is a major health concern worldwide. It is the second most common cancer with high morbidity and mortality. Its mortality rate can be reduced by early detection through CRC screening and detection, as it has a good prognosis in the early stages. The results of this study highlighted several critical points regarding the knowledge, attitudes, and practices of healthcare personnel in KP, Pakistan, toward CRC screening. In the study by Yau et al. (2019), 43 (21.8%) participants were male and 154 (78.2%) were female. By contrast, our study included 137 (83.5%) males and 127 (16.5%) females [4]. Despite high awareness of the screening methods, only 6.1% of the participants routinely recommended CRC screening for all patients, with 22.6% recommending it occasionally and 71.3% rarely or never recommending it. Despite this, almost all participants had a positive attitude toward CRC screening and were interested in training if provided. According to Yau et al. (2019), 69.9% of primary care physicians (PCPs) reported practicing CRC screening. However, only 25.9% of these PCPs screened over 50% of all eligible patients. In comparison, in the United States, almost 99% of PCPs practiced CRC screening, while in Italy, about 80% of physicians practiced the same [4,10,11]. By contrast, our study found that only 6.1% of the participants routinely recommended CRC screening for all patients, 22.6% recommended it occasionally, and 71.3% rarely or never recommended it.

Mohamed et al. (2020) reported that the majority (90.6%) of physicians mentioned that they would recommend CRC screening for their asymptomatic patients [12]. This significant disparity underscores the critical need for improved education and training among healthcare professionals in our study area to enhance the implementation of CRC screening practices. According to a study conducted in Ghana (Africa), about 10% of physicians would recommend CRC screening for asymptomatic, average-risk patients who met the age inclusion criteria [13]. According to Pinar et al. (2020), about 68.4% of participants were aware that colonoscopy is used for CRC screening. By contrast, our study found a higher awareness level, with 151 (92.1%) of participants acknowledging colonoscopy as a CRC screening method [14]. Lussiez et al. (2020) reported that 9.6% of participants did not recommend CRC screening for average-risk, asymptomatic patients. By contrast, our study found that a significantly higher proportion, 71.3%, rarely or never recommended CRC screening [13]. Lopez et al. (2013) reported that 24.4% of participants recommended colonoscopy for CRC screening, while 2.5% did not recommend it. By contrast, our study found that only 6.1% of participants routinely recommended CRC screening for all patients, 22.6% recommended it occasionally, and 71.3% rarely or never recommended it [15]. Lussiez et al. (2020) reported that 86.2% of participants identified patients' lack of awareness of screening and not perceiving CRC as a serious health threat as a barrier to CRC screening, while 85.7% noted a lack of insurance coverage. Obada et al. (2022) reported that 92.3% of people lacked awareness about CRC screening, and 15.9% of physicians lacked awareness. In our study, 76.2% of the participants cited a lack of awareness as a significant barrier, 29.9% mentioned reimbursement issues, and 44.5% identified a lack of awareness among physicians [13,16].

Perin et al. (2015) reported that 40% of participants were aware of colonoscopy, 77% were aware of FOBT, and 51% were aware of sigmoidoscopy for CRC screening. Similarly, Jenn et al. (2011) found that 87% of the participants recognized FOBT as a screening method for CRC. Meanwhile, Thanapirom et al. (2012) reported colonoscopy as the most popular tool used for CRC screening (47.5%), followed by FOBT (40.6%). By contrast, our study found higher awareness levels among healthcare personnel: 92.1% were aware of colonoscopy, 65.9% of FOBT, and 48.2% of flexible sigmoidoscopy for CRC screening [17-19].

Our study revealed a significant knowledge gap among healthcare professionals regarding CRC screening. While the majority of senior healthcare workers were familiar with screening guidelines and modalities, many junior healthcare workers lacked this knowledge. A significant barrier to routine screening was a lack of familiarity with CRC screening guidelines, with only 16.5% being fully familiar and 64% not familiar at all. Major factors influencing the recommendation of CRC screening included family history of CRC, patient age, availability of screening facilities, patient's overall health status, and patient preference. Key barriers identified were a lack of patient awareness, limited access to screening facilities, lack of physician awareness, reimbursement issues, and time constraints during patient visits [20]. Key barriers to early screening and diagnosis identified by physicians included a lack of patient awareness (76.2%, n = 125), screening facility limitations (66.5%, n = 109), and a lack of awareness among healthcare workers (44.5%, n = 73). Similarly, a study conducted in Ghana found that major barriers identified by physicians (over 85%) were a lack of patient awareness, as patients did not perceive CRC as a serious health concern, and high screening costs [13]. The strength of our study lies in its comprehensive assessment of the knowledge, practice, and attitudes of healthcare personnel across five tertiary care hospitals in KP, Pakistan. This is particularly notable as there were no previous studies on this topic in Pakistan. Our findings underscore the need for targeted education and training programs to bridge the knowledge gap and enhance adherence to CRC screening guidelines, ultimately improving patient outcomes.

The findings recommend that proper training for healthcare personnel is required and should stay updated on CRC screening guidelines and routinely recommend CRC screening for appropriate patients. Awareness sessions and screening facilities are also required. More healthcare providers from different departments and more healthcare systems within KP studies are necessary.

## Conclusions

CRC prevention and control are crucially dependent on effective health education initiatives and efficient screening programs. Our study emphasizes the importance of enhancing healthcare personnel's knowledge and awareness of CRC detection methods and screening guidelines. It is imperative to ensure that healthcare professionals stay updated on critical health issues, such as early CRC detection, as their awareness and prompt recommendation of screening can potentially reduce the mortality and morbidity associated with this disease. By implementing comprehensive education and training programs, we can empower healthcare providers to play a pivotal role in promoting early detection and improving patient outcomes in CRC management. Moreover, early diagnosis significantly improves the prognosis of patient treatment.

## Appendices

Study questionnaire

This is a descriptive, cross-sectional study. It is a part of a research project focusing on awareness about colorectal carcinoma and its screening. All the information will be presented in research (if so), and a number will be assigned to it and not your actual name. Please proceed if you agree to participate in this research. By starting, you give consent to participate, but you are free to withdraw at any stage without giving any reason.

* Indicates a required question 

1. What is your age? 

o 24-26 

o 27-29 

o 30-32 

o 33-35 

o 36-38 

o older than 40 

2. What is your name? 

Your answer 

3. At which post are you working currently? 

o Professor 

o Ass. Professor 

o Training Medical Officer 

o House Officer 

o Medical Officer 

4. Years of experience as a doctor 

Your answer:

5. In which hospital are you working currently? 

Your answer:

6. What is your gender? 

o Male 

o Female 

o transgender 

7. How familiar are you with the term "colorectal carcinoma screening"?

o very familiar 

o somewhat familiar 

o not familiar at all 

8. What methods of colorectal carcinoma screening are you aware of? (Select all that apply)* 

o Colonoscopy 

o Fecal occult blood test (FOBT) 

o Flexible sigmoidoscopy 

o Virtual colonoscopy 

o Stool DNA test (FIT-DNA test) 

9. How often do you recommend colorectal carcinoma screening to your patients?

o Routinely (for all eligible patients) 

o Occasionally (for some patients) 

o Rarely or never

10. What factors influence your decision to recommend colorectal carcinoma screening? (Select all that apply) * 

o Age of the patient 

o Family history of colorectal cancer 

o Patient's overall health status 

o Patient's preferences 

o Availability of screening facilities 

11. Are you familiar with the current colorectal carcinoma screening guidelines?

o Yes, I am familiar with the guidelines 

o I am somewhat familiar with the guidelines 

o No, I am not familiar with the guidelines 

12. How confident do you feel in your ability to discuss the benefits and risks of colorectal carcinoma screening with your patients? 

o Very confident 

o Moderately confident 

o Not confident at all 

13. Have you received any specific training or education regarding colorectal carcinoma screening? 

o Yes 

o No 

14. How often do you update your knowledge about colorectal carcinoma screening guidelines and recommendations? 

o Regularly (e.g., attending conferences, reading journals) 

o Occasionally 

o Rarely or never 

15. What sources do you typically rely on to stay updated about colorectal carcinoma screening? (Select all that apply) * 

o Medical journals 

o Clinical practice guidelines 

o Continuing medical education (CME) courses 

o Colleagues or peers 

o Online resources 

o Other: 

16. In your opinion, what are the barriers or challenges in implementing colorectal carcinoma screening effectively? * 

o Lack of patient awareness 

o Lack of physician knowledge or training 

o Limited access to screening facilities 

o Time constraints during patient visits 

o Reimbursement issues 

17. Would you be interested in receiving additional education or resources to improve your knowledge and practice regarding colorectal carcinoma screening?

o Yes, definitely 

o Maybe 

o No, not interested 

18. Any additional comments or suggestions regarding colorectal carcinoma screening awareness among doctors? 

Your answer:
